# The effect of dolutegravir on the pharmacokinetics of metformin in healthy subjects

**DOI:** 10.7448/IAS.17.4.19584

**Published:** 2014-11-02

**Authors:** Jian Zong, Julie Borland, Fred Jerva, Brian Wynne, Mike Choukour, Ivy Song

**Affiliations:** 1 Clinical Pharmacology Modeling & Simulation, GlaxoSmithKline, RTP, USA; 2 CPSSO, GlaxoSmithKline, RTP, USA; 3 Infectious Disease MDD, GlaxoSmithKline, RTP, USA; 4 Biostatistics, Parexel, Sarasota, FL, USA

## Abstract

**Introduction:**

Dolutegravir (DTG) is an HIV integrase strand transfer inhibitor approved for use in combination with other antiretrovirals for the treatment of HIV-infection in adults and adolescents. Metformin is a drug frequently used in diabetic HIV-infected patients, which requires titration to optimize dosing. *In vitro*, DTG inhibits organic cation transporter 2 (OCT2) and multidrug and toxin extrusion transporter 1 (MATE 1) which are known to be involved in the disposition of metformin. The objective of this study was to assess the drug interaction between DTG and metformin.

**Materials and Methods:**

This was an open-label, parallel-group, three-period crossover study in healthy adult subjects. Eligible subjects were enrolled into one of the two treatment cohorts (15 subjects/cohort). Subjects received metformin 500 mg q12h for 5 days in Period 1; metformin 500 mg q12h plus DTG 50 mg q24h (Cohort 1) or 50 mg q12h (Cohort 2) for 7 days in Period 2; and metformin 500 mg q12h for 10 days in Period 3. There were no washout periods between treatments. All doses of study drug were taken with a moderate-fat meal. Serial plasma PK samples and safety assessments were obtained throughout the study. Non-compartmental PK analysis was performed and geometric least squares (GLS) mean ratios and 90% confidence intervals (CI) were generated by the mixed effect model for within-subject treatment comparisons for each cohort.

**Results:**

Fourteen and thirteen subjects completed study in Cohort 1 and Cohort 2, respectively. Plasma exposures of metformin were significantly increased when co-administered with DTG (Table 1).

There were no apparent changes in metformin half-life and tmax. Increased metformin plasma exposure returned to normal levels observed in Period 1 after DTG was discontinued in Period 3. No Grade 3 or 4 adverse events (AEs), deaths or serious AEs were reported during the study. Most frequently reported drug-related AEs were headache (9), loose stools (8), and nausea (7). All AEs were mild or Grade 1 with the exception of one Grade 2 headache.

**Conclusions:**

Co-administration of DTG and metformin was well tolerated, yet significantly increased metformin plasma exposure; effects were DTG dose dependent. Though metformin has a wide therapeutic index and alone is not associated with hypoglycemia, close monitoring is recommended when co-administering metformin and DTG. Dose adjustments of metformin may be considered.

**Table 1:** Statistical comparison of metformin PK parameters with and without dolutegravir

	GLS mean		GLS mean ratio (90% CI)
Plasma Metformin PK Parameter	Metformin Alone (Period 1)	Metformin+DTG (Period 2)	Metformin+DTG vs. Metformin Alone
Cohort 1 (DTG 50 mg QD)	n=15	n=14	
Cmax (µg/mL)	0.932	1.55	1.66 (1.53, 1.81)
AUC(0-τ) (hr*µg/mL)	6.83	12.2	1.79 (1.65, 1.93)
Cohort 2 (DTG 50 mg BID)	n=15	n=14	
Cmax (µg/mL)	0.845	1.878	2.11 (1.91, 2.33)
AUC(0-τ) (hr*µg/mL)	6.49	15.9	2.45 (2.25, 2.66)

